# Molecular Basis of Hydatidiform Moles—A Systematic Review

**DOI:** 10.3390/ijms25168739

**Published:** 2024-08-10

**Authors:** Shadha Nasser Mohammed Bahutair, Rajani Dube, Manjunatha Goud Bellary Kuruba, Rasha Aziz Attia Salama, Mohamed Anas Mohamed Faruk Patni, Subhranshu Sekhar Kar, Rakhee Kar

**Affiliations:** 1 Department of Obstetrics and Gynecology, RAK College of Medical Sciences, RAK Medical & Health Sciences University, Ras al Khaimah P.O. Box 11172, United Arab Emirates; shadha@rakmhsu.ac.ae; 2 Department of Biochemistry, RAK College of Medical Sciences, RAK Medical & Health Sciences University, Ras al Khaimah P.O. Box 11172, United Arab Emirates; manjunatha@rakmhsu.ac.ae; 3 Department of Community Medicine, RAK College of Medical Sciences, RAK Medical & Health Sciences University, Ras al Khaimah P.O. Box 11172, United Arab Emirates; rasha.aziz@rakmhsu.ac.ae (R.A.A.S.); mohamedanas@rakmhsu.ac.ae (M.A.M.F.P.); 4 Department of Public Health and Community Medicine, Kasr El Ainy Faculty of Medicine, Cairo University, Cairo 12613, Egypt; 5 Department of Pediatrics, RAK College of Medical Sciences, RAK Medical & Health Sciences University, Ras al Khaimah P.O. Box 11172, United Arab Emirates; subhranshu.kar@rakmhsu.ac.ae; 6 Department of Pathology, Jawaharlal Institute of Postgraduate Medical Education & Research, Puducherry 605006, India; drrakheekar@gmail.com

**Keywords:** gestational trophoblastic disease, hydatidiform mole, complete molar pregnancy, partial molar pregnancy, choriocarcinoma, molecular basis of molar pregnancy

## Abstract

Gestational trophoblastic diseases (GTDs) encompass a spectrum of conditions characterized by abnormal trophoblastic cell growth, ranging from benign molar pregnancies to malignant trophoblastic neoplasms. This systematic review explores the molecular underpinnings of GTDs, focusing on genetic and epigenetic factors that influence disease progression and clinical outcomes. Based on 71 studies identified through systematic search and selection criteria, key findings include dysregulations in tumor suppressor genes such as *p53*, aberrant apoptotic pathways involving *BCL-2* (B-cell lymphoma), and altered expression of growth factor receptors and microRNAs (micro-ribose nucleic acid). These molecular alterations not only differentiate molar pregnancies from normal placental development but also contribute to their clinical behavior, from benign moles to potentially malignant forms. The review synthesizes insights from immunohistochemical studies and molecular analyses to provide a comprehensive understanding of GTD pathogenesis and implications for personalized care strategies.

## 1. Introduction

**Figure 1:**
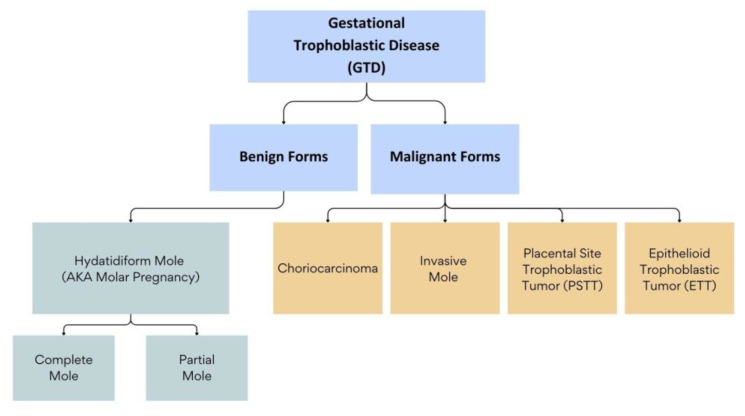
Classification of Gestational Trophoblastic Diseases (GTDs).

Gestational trophoblastic diseases (GTDs) encompass a spectrum of conditions that range from benign to malignant forms and are characterized by abnormal growth of trophoblastic cells. The benign forms of GTD comprise molar pregnancies, which include complete hydatidiform moles (CHMs) and partial hydatidiform moles (PHMs). These two entities have varying clinical presentations and outcomes [1]. The malignant forms of GTD, also known as gestational trophoblastic neoplasms (GTNs), include choriocarcinoma (CC) and invasive moles (IMs), epithelioid trophoblastic tumors (ETTs), and placental-site trophoblastic tumors (PSTTs). These neoplasms can arise de novo after a normal pregnancy, or, most commonly, following a molar pregnancy [2] (Figure 1).

Molar pregnancies typically result from errors in gamete formation and the fertilization process, which lead to the formation of an abnormal zygote with an abnormal amount of parental genetic material contributions. In CHM, the total amount of genetic material is normal, but all chromosomes are paternal without maternal chromosomes [2]. In CHM, an enucleated egg is either fertilized by one sperm, whose genetic material then duplicates (more common), or is fertilized by two sperms (less common). The result is a diploid zygote with 46 paternal chromosomes and no maternal genetic material (i.e., androgenetic diploid) [3]. However, there is a subtype of CHM called familial recurrent hydatidiform moles (FRHMs), in which cells are biparental diploid (i.e., cells contain 23 paternal chromosomes and 23 maternal chromosomes) [4,5]. This entity will be discussed in detail later in this review. In PHM, a normal egg (1n) is fertilized by more than one sperm, resulting in polyploidy [3]. Usually, there is triploidy, with an extra paternal haploid set of chromosomes, with the most common type being 69, XXX (90% of cases) [6].

The incidence of molar pregnancies varies across different world regions, ranging from 0.2 to 9.9 per 1000 pregnancies. However, its incidence is higher in Asian and African ethnicities [7]. The possibility of developing post-molar GTN is the primary reason for extended follow-up with serial beta-human chorionic gonadotropin (β-HCG) monitoring in patients after the evacuation of molar pregnancies. The incidence of post-molar pregnancy is around 15–20% in CHM but is only 0.5–5% in PHM [8,9]. This discrepancy is the reason follow-up protocols after the evacuation of a molar pregnancy differ for each subtype. Thus, it is essential to differentiate between the two entities during histopathological testing. The diagnosis of molar pregnancy follows specific morphological criteria through histopathological examination. The microscopic appearance differs between CHMs, PHMs, and hydropic abortions (HAs) [10]. However, challenges arise in differentiating between CHM and PHM, and even between molar pregnancy and HAs, due to overlapping histological signs, especially in the early stages of pregnancy [11,12]. The advent of ultrasonographic machines has made it possible to diagnose molar pregnancies in the early stages, which has paradoxically led to a more confusing morphological assessment and sometimes erroneous interpretation of tissue specimens during histopathological examination [11,12]. Multiple ancillary techniques utilizing immunohistochemical staining and genetic analyses have emerged to help differentiate between CHMs, PHMs, and HAs [13].

**Figure 2:**
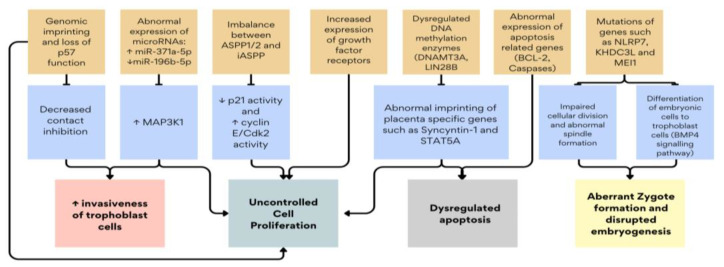
Summary of the key molecular dysregulations in GTD. ↑: increased gene expression or protein/enzyme activity, ↓: decreased gene expression or protein/enzyme activity.

Research into these ancillary techniques has shed light on the intricate molecular mechanisms driving molar pregnancies. These include dysregulations in tumor suppressor genes such as *p53*, apoptotic pathways involving BCL-2 (B-cell lymphoma-2), and aberrant expression of growth factor receptors and microRNAs (micro-ribose nucleic acid) [14,15]. These molecular alterations not only differentiate molar pregnancies from normal placental development but also contribute to their clinical behavior, ranging from benign moles to potentially malignant forms [16,17,18]. Figure 2 summarizes the key molecular mechanisms described in this review. This review explores the current understanding of molecular markers and genetic mutations in molar pregnancies, emphasizing their roles in cell proliferation, apoptosis, and trophoblastic differentiation. By synthesizing findings from immunohistochemical studies and molecular analyses, this article aims to provide insights into the pathogenesis of GTDs.

## 2. Materials and Methods

### 2.1. Search Strategy

A systematic search was conducted across electronic databases including PubMed, Scopus, and CINAHL EBSCO. Medical Subject Headings (MeSH) terms and free-text keywords such as “Hydatidiform mole”, “gestational trophoblastic disease”, and “molar pregnancy” were used in conjunction with “gene”, “genome”, “genetic”, “immunohistochemistry” or “molecular” in February 2024 to retrieve relevant data from January 2004 to January 2024. Additionally, the references for pertinent studies were manually searched if they were not included in these databases.

### 2.2. Eligibility Criteria

#### 2.2.1. Inclusion Criteria

Studied were included if they met all the following criteria—1, 2, and 3:

1. Articles written in the English language or an English translation is available;
2. Full-text articles reporting on human genes, genome, genetics, or molecular bases;
3. Articles containing information on gestational trophoblastic disease, hydatidiform mole, or molar pregnancy.

All eligible studies published between January 2014 and January 2024 were included for review. No retracted papers were included in this review.

#### 2.2.2. Exclusion Criteria

Exclusion criteria included duplicate studies, review articles, non-genetic and non-immunohistochemical studies, articles not available in the English language, and studies where full-text articles were only available with payment. Conference abstracts, expert opinions, and critical appraisals were also excluded from the review.

### 2.3. Study Selection

After a comprehensive search of the databases, a total of 5259 results were initially retrieved. All the abstracts and study titles were screened, and duplicates were removed. Furthermore, there were a total of 5188 studies excluded, as they either did not fit the inclusion criteria, were animal studies, included only gestational trophoblastic neoplasia, placental abnormalities other than hydatidiform mole, or did not explore the genetic basis of the disease. Eventually, 71 articles met all the criteria and were included in the review. A summary of the studies included in this review is available as Supplementary Material Table S1.

### 2.4. Data Collection

**Figure 3:**
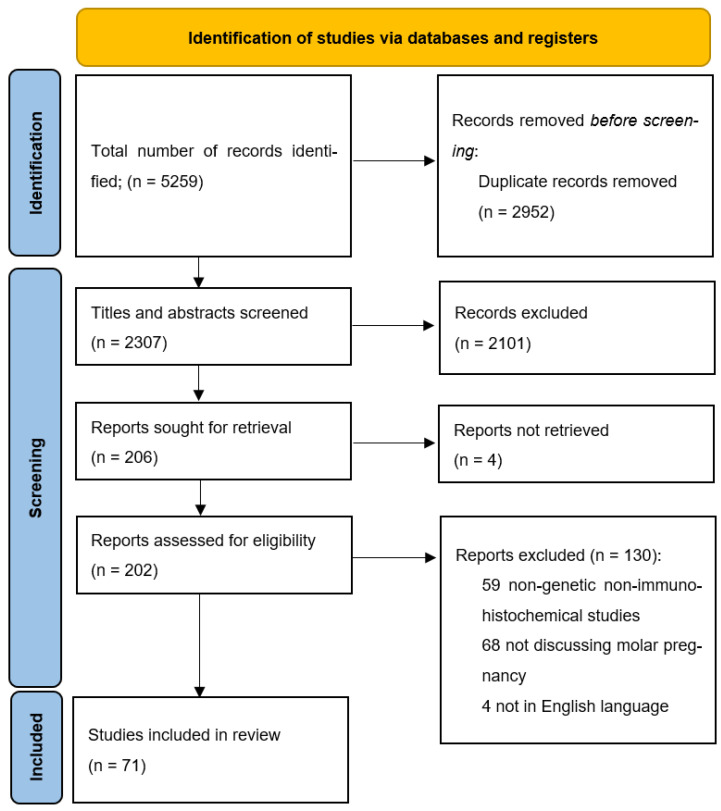
PRISMA flow diagram for study inclusions.

All authors (SB, RD, MA, MG, RS, RK, and SSK) independently reviewed all titles. The potential relevance of studies for inclusion in the review was determined through discussion. Selected titles and abstracts were further screened to avoid overlap of cases. Full-text copies of the selected papers were obtained, and the same reviewers independently extracted relevant data regarding study characteristics, results of molecular testing, and significant associations with GTD outcomes. Whenever possible, single case reports were cross-checked with other reports from the same location and hospital. Finally, studies were screened by assessing their suitability for inclusion in the evidence acquisition for the molecular basis of hydatidiform mole. Figure 3 illustrates the Preferred Reporting Items for Systematic Reviews and Meta-Analysis (PRISMA).

## 3. Results and Discussion

### 3.1. Genomic Imprinting

Genomic imprinting, regulated by DNA (deoxyribose nucleic acid) methylation at differentially methylated regions (DMRs), governs the expression of genes based on their parental origin [19]. In complete moles, which are predominantly androgenetic diploids, the absence of maternal genomic contribution results in significant implications for imprinted genes such as *p57kip2* (p57 cyclin-dependent kinase inhibitor 2) [5,6,20]. *P57kip2*, paternally imprinted and expressed exclusively from the maternal allele, exhibits markedly reduced expression in CHMs compared to partial moles and normal placentas [21,22,23,24,25,26,27]. This distinct immunohistochemical pattern of *p57kip2* has become crucial for distinguishing between different types of hydatidiform moles [11,12,28]. Studies using human trophoblast stem cells have highlighted *p57kip2’s* role in regulating contact inhibition, suggesting that its diminished expression contributes to the uncontrolled proliferation characteristic of CHMs [29]. Despite rare instances of biparental diploid CHMs [5,30] and retained maternal copy of respective chromosomes [31], the majority exhibit abnormal genomic imprinting and epigenetic dysregulation, including down-regulation of DNMT3A (DNA Methyltransferase 3 Alpha) and LIN28B (Lin-28 Homolog B), key enzymes for DNA methylation and parental imprinting maintenance [32,33,34]. Sanchez-Delgado et al. confirmed abnormalities in the epigenomic regulation of placenta-specific maternally inherited genes, characterized by atypical methylation patterns [35]. Abnormal methylation patterns affect genes like *ERVWE1* (Syncyntin-1), implicated in syncytiotrophoblast function and apoptosis regulation, further underlining the impact of epigenetic changes in CHM pathogenesis [36,37]. Abnormal methylation affects other genomic components, including LINE-1 (Long Interspersed Nuclear Element-1), a transposable element, and the gene *STAT5A* [38,39]. While typically *p57*-negative, rare cases of CHM show aberrant *p57* expression linked to retained maternal chromosomes or biparental diploidy, emphasizing the utility of genotyping in diagnosing and understanding the molecular basis of molar pregnancies [40,41,42,43]. Similarly, although partial hydatidiform moles are typically *p57*-positive, rare instances (1.3%) have been reported with negative *p57* results [44]. Genomic imprinting in gametogenesis is responsible for genetic expression in the offspring depending on parent-of-origin, and the human placenta has a high and prolific expression of imprinted genes [45,46]. The genotype of molar pregnancies, particularly heterozygous/dispermic complete moles, correlates with a higher risk of developing post-molar gestational trophoblastic neoplasia, suggesting a potential role for additional paternal imprints in promoting trophoblastic proliferation [16]. Further research is needed to elucidate the precise mechanisms underlying these genomic imprinting abnormalities and their clinical implications in molar pregnancy pathogenesis [1,2,3,4]. 

### 3.2. Recurrent Hydatidiform Mole

Recurrent hydatidiform moles (RHMs) encompass two distinct categories: sporadic cases and familial forms. Sporadic RHM typically occurs with a recurrence rate of 1–6% among individuals with a history of molar pregnancy [47]. These cases often exhibit a monospermic or dispermic androgenetic diploid genotype [5]. In contrast, cases of familial RHM (FRHM) are generally biparental diploids and characterized by two or more affected female family members experiencing recurrent molar pregnancies [4,5]. These familial cases are considered autosomal recessive inherited disorders and are associated with abnormal CpG methylation in imprinted genes [48]. 

The predominant genetic culprits linked to FRHM include mutations in *NLRP7* (Nucleotide-Binding Oligomerization Domain, Leucine Rich Repeat and Pyrin Domain-Containing 7) and *KHDC3L* (KH Domain Containing 3 Like), with *NLRP7* mutations found in 40–80% and *KHDC3L* mutations in 10–14% of affected individuals [47,49]. A mutation analysis by Nguyen et al. of 113 patients with recurrent molar pregnancies showed that mutations of *NLRP7* and *KHDC3L* were associated with diploid biparental HM, while recurrent molar pregnancies without mutations were associated mostly with diploid androgenic monospermic and triploid biparental dispermic [50].

Multiple case reports and case series identified mutations in the homozygous state or combined heterozygous states involving the *NLRP7* and *KHDC3L* genes associated with recurrent hydatidiform moles [48,51,52,53,54,55,56]. *NLRP7*, located on chromosome 19q13.4, is a maternal effect gene, which means that the phenotype is influenced by mutations in maternal genes only, crucial for oocyte and embryo development [57]. It localizes in the cytoskeleton, which contains microtubules essential for cellular division [57]. Homozygous mutations in *NLRP7* disrupt embryo development by impairing cellular division and organization within the cytoskeleton, leading to developmental arrest and failed embryo progression. In a study investigating 10 embryos from a woman with recurrent hydatidiform mole and a homozygous pathogenic variant in *NLRP7* (c.2810+2T>G), all embryos exhibited developmental arrest and did not advance to a stage suitable for transfer [58]. This mutation primarily affects embryos when inherited maternally, owing to its maternal inheritance pattern [48]. Additionally, *NLRP7* interacts with *YY1* (Yin Yang 1) to activate the BMP4 (Bone Morphogenetic Protein 4) signaling pathway, stimulating embryonic cells to differentiate into trophoblasts rather than maintaining pluripotency to differentiate into various cell types, thereby contributing to mole formation [49,59]

*KHDC3L*, though less frequently implicated in biparental complete hydatidiform mole (BICHM), likely forms complexes with *NLRP7* to assist in oogenesis and early embryonic development [39]. Mutations in these genes can dysregulate inflammatory cytokine secretion within the endometrial cavity, potentially altering the implantation microenvironment and contributing to molar pregnancy pathogenesis [39]. Specifically, mutations in *NLRP7* have been associated with lower levels of immune signaling molecules like IL-1β [60]. The altered immune function observed in patients with *NLRP7* mutations may be relevant to understanding the pathogenesis of molar pregnancy. Reddy et al. identified 11 *NLRP7* variants in homozygous or compound heterozygous state in individuals with a history of recurrent hydatidiform mole. Some of these variants were associated with abnormalities in transcription and with post-transcriptional modifications of mRNA (messenger ribose nucleic acid) that affect splicing and result in either the absence of gene transcription or the formation of abnormally long or truncated proteins [61]. Reddy et al.-identified *NLRP7* variants along with other variants associated with RHM are presented in Table 1 [47,48,52,53,54,55,56,58,61,62,63,64,65,66,67,68]. 

NM et al. examined the interaction of the *p57KIP2* gene with *NLRP7* mutations in hydatidiform mole (HM). Analyzing 36 products of conception (POCs) from patients with two defective *NLRP7* alleles revealed that all samples were diploid biparental [69]. They found that the expression of *p57KIP2* varied based on the type of *NLRP7* mutation: missense mutations were associated with positive *p57KIP2* expression and features similar to partial HMs, while protein-truncating mutations correlated with negative *p57KIP2* expression and characteristics of complete HMs [69]. These results suggest that *NLRP7* mutations impact trophoblast cell differentiation and proliferation to varying degrees, with severe mutations leading to excessive proliferation and mild mutations allowing some differentiation.

In addition to *NLRP7* and *KHDC3L*, other gene variants have been identified in RHM cases, such as *MEI1* (meiotic double-stranded break formation protein 1), *TOP6BL/C11orf80* (type 2 DNA topoisomerase 6 subunit B-like), and *REC114* (Recombination Protein 114), all of which affect oocyte meiosis and early embryonic development [67]. These mutations can result in abnormal spindle morphology, misaligned chromosomes, extrusion of all chromosomes into the polar body, and other defects that can lead to empty oocytes, androgenetic zygote formation, and subsequent molar pregnancy [67].

Understanding the genetic underpinning of diseases through comprehensive mutation analysis provides insights into the complex mechanisms involved [70] and has the potential to offer avenues for personalized care in affected individuals. Similarly, the genetic and molecular basis of different molar pregnancies has a pivotal role in decisions about treatment protocols and follow-ups. 

### 3.3. Molecular Dysregulations in GTD

Immunohistochemical studies on hydatidiform moles offer valuable insights into the molecular underpinnings that distinguish them from normal placentas and hydropic abortions. These investigations reveal significant alterations in markers crucial for cell cycle regulation, apoptosis, and trophoblast differentiation, shedding light on the pathogenesis of molar pregnancies. Table 2 summarizes the molecular dysregulations associated with Gestational Trophoblastic Disease [14,68,71,72,73,74,75,76,77,78,79,80,81,82,83,84,85]. 

The expression of *p53* in cytotrophoblasts is markedly elevated in molar pregnancies compared to hydropic abortions [14,80,81]. Studies indicate higher levels of *p53* expression in patients with invasive moles and choriocarcinoma compared to those with non-invasive hydatidiform moles [86,87,88]. Hadi et al. demonstrated that more than 55% positive staining for the *TP53* gene can effectively distinguish non-invasive hydatidiform mole from invasive forms and choriocarcinoma with 100% sensitivity and 92.9% specificity, albeit based on a small sample size [88]. 

*P53*, recognized as a tumor suppressor gene and often referred to as the “guardian of the genome”, is normally expressed in cytotrophoblasts and infrequently in stromal cells [81]. Its primary functions include inducing cell cycle arrest and apoptosis [81,89]. This is achieved through the transcriptional activation of p21/WAF1, which interacts with cyclin E/Cdk2 and cyclin D/Cdk4 complexes, leading to G1 arrest in the cell cycle [89]. Therefore, *p53* serves as a marker of proliferative activity, crucial for regulating proliferation by inducing either cell cycle arrest or apoptosis. The overexpression of *p53* in molar tissues reflects the heightened proliferative capacity of trophoblastic cells [81]. Similarly, Studies have demonstrated significantly higher expression of *p63*, a tumor suppressor gene from the *p53* family, in molar pregnancies compared to hydropic abortions (*p*-value < 0.05) [72,83].

The *ASPP* (Ankyrin-repeat, SH3-domain, and proline-rich region containing protein) family, including *ASPP1* and *ASPP2*, modulates p53 activity and is found to be dysregulated in gestational trophoblastic diseases (GTD) [90,91]. Normally functioning as tumor suppressors, *ASPP1* and *ASPP2* stimulate p53-mediated transcriptional modifications of p21. However, Mak et al. have reported their downregulation in GTD [90,91]. Another crucial member of this family is *iASPP* (inhibitory ASPP), which exhibits increased expression in complete hydatidiform mole and choriocarcinoma compared to normal placental tissue [92]. Chan et al. demonstrated that silencing *iASPP* was associated with reduced production of autophagy-related proteins (LC3) and increased susceptibility to oxidative stress in choriocarcinoma cells [92]. These findings underscore the role of imbalanced expression of *ASPP1/2* (downregulation) and *iASPP* (upregulation) in the pathogenesis of GTD [90,91,92]. 

Several mutations in *p53* genes have also been identified in patients with molar pregnancy. Chan et al. identified two missense mutations (p.R249S and p.R248Q) that disrupt *p53* DNA binding sites, impairing its ability to control cell proliferation [93]. Additionally, a nonsense mutation (p.R213X) was reported to prematurely truncate the protein, resulting in loss of its normal function [93]. Several studies showed that *Ki-67* (Kiel-67) exhibits heightened expression levels in hydatidiform moles compared to both hydropic abortions and normal placental tissues [72,77]. This protein plays a crucial role in trophoblast differentiation and is primarily expressed by cytotrophoblasts. Notably, studies have shown significant overexpression of *Ki-67* in CHM when analyzed in conjunction with Cyclin E, a key promoter of cell cycle progression, demonstrating a statistically significant difference compared to normal and hydropic placentas (*p*-value < 0.05) [94]. 

Apart from aberrant cell proliferation, dysregulation of apoptosis also plays a pivotal role in the pathogenesis of GTD. Studies examining the apoptotic index in molar pregnancies yield disparate findings, primarily due to methodological variations in apoptosis assessment. One study focused on caspase-3, a critical enzyme in caspase-dependent apoptosis, and reported diminished expression levels in GTN, suggesting reduced apoptosis [95]. In contrast, another study utilizing the TUNEL assay (terminal deoxynucleotidyl transferase-mediated deoxyuridine triphosphate nick end labeling), which detects DNA fragmentation as a late-stage apoptosis marker, indicated heightened apoptotic activity in GTD [96]. Furthermore, a study comparing the expression of caspases between persistent CHM and regressed CHM found no significant difference in apoptosis levels, though it was limited by a small sample size [97]. These findings suggest that while apoptosis levels are elevated in GTD, it may predominantly occur through caspase-independent pathways rather than the traditional caspase-dependent pathway. This underscores the necessity for further research to elucidate the specific apoptotic mechanisms at play in molar pregnancies and their clinical implications. 

*BCL-2*, an anti-apoptotic gene, plays a crucial role in regulating caspase-dependent apoptosis, and acts as an anti-proliferative protein by inhibiting cell transition from quiescence to the S-phase [98]. In the placenta, *BCL-2* expression in syncytiotrophoblasts controls apoptosis in these multinuclear cells. It prevents the spread of apoptotic changes and the fragmentation of other nuclei sharing the same cytoplasm when apoptosis occurs in one nucleus of the syncytiotrophoblast [96]. Studies assessing *BCL-2* expression in molar pregnancies have yielded conflicting results. Several studies have demonstrated significantly reduced BCL-2 immunohistochemical staining in the syncytiotrophoblasts of complete mole compared to control specimens [68,71,73,99]. Lin et al. demonstrated that CHM progressing to gestational trophoblastic neoplasia expressed higher levels of miR-181b-5p and miR181d-5p and lower levels of their target, *BCL-2*, compared to those that regressed following evacuation [100]. Conversely, other studies have reported increased *BCL-2* expression in molar pregnancies or found insignificant staining differences between molar and non-molar placentas [72,101,102]. Missaoui et al., in their evaluation of 220 specimens classified based on morphological appearance and molecular markers into 140 CHMs, 41 PHMs, and 39 HAs, found significantly higher *BCL-2* immunostaining in partial mole (61%) and CHM (73.6%) compared to hydropic abortions (7.7%, *p* = 0.001, *p* < 0.0001 respectively), which may explain earlier findings of reduced caspase expression in GTD [87,89]. Larger studies incorporating reliable diagnostic criteria and ploidy analysis are warranted to accurately delineate the alterations in *BCL-2* expression in molar pregnancy. In contrast, the immunoexpression of Bax, another regulator gene in the apoptosis pathway, did not show significant differences between molar pregnancies and non-molar pregnancies according to Reza et al. [71]. 

The molecular distinctions between different types of GTDs are crucial in identifying markers for the behavior and aggressiveness of molar pregnancies. Research has identified distinct patterns of growth factor receptor expression in various trophoblast types. Specifically, *EGFR* ( Epidermal Growth Factor Receptor) and *ERBB4* (Erythroblastic Leukemia Viral Oncogene Homolog 4) are notably overexpressed in actively proliferating trophoblasts [16]. Additionally, trophoblasts exhibiting invasive characteristics demonstrate heightened expression of *ERBB2*, also known as *HER2*/*neu* (Human Epidermal Growth Factor Receptor 2) and *C-erbB-2* (Cellular Erythroblastic Leukemia Viral Oncogene Homolog 2), and *ERBB3* [103,104]. Epidermal Growth Factor (EGF) and Heparin-Binding EGF-like Growth Factor (HB-EGF) enhance cell signaling pathways in *EGFR*-expressing trophoblasts, thereby promoting cell cycle progression and contributing to proliferative activity in hydatidiform moles [16]. 

*C-erbB-2* was also found by Erol et al. to be significantly overexpressed in complete moles compared to partial moles and hydropic abortions (HAs) [73]. Previous studies suggest that this overexpression may correlate with aggressive behavior in CHM [17,18]. Another tyrosine receptor kinase, CD117 (c-KIT), presents on various cell types including mast cells, hematopoietic stem cells, and germ cells [105], exhibits increased activation in molar tissues compared to hydropic abortions, and is associated with aggressive CHM behavior [73]. Activation of CD117 through stem cell factor (SCF) regulates processes such as proliferation, cell differentiation, apoptosis, and cell adhesion [105,106]. 

miRNAs have been implicated in GTDs based on their distinct expression profiles in trophoblastic tissues. Among these, miR-371a-5p is notable for its oncogenic properties in various cancers and is found to be upregulated in progressive CHMs, suggesting a potential role in the progression of malignancy [15]. Another miRNA, miR-196b-5p (miR-196b), functions as a tumor suppressor and exhibits reduced expression in CHM tissues compared to normal placentas [78]. This decrease correlates with elevated levels of Mitogen-activated protein kinase 1 (*MAP3K1*), a protein promoting cell proliferation and differentiation [78]. Increasing miR-196b expression in hydatidiform mole cells has been shown to diminish their proliferation and invasion, suggesting its potential as both a diagnostic marker and therapeutic target [78]. Furthermore, miR-21 is significantly overexpressed in tissues from hydatidiform moles, where it enhances aggressive behaviors such as proliferation, migration, and invasion of trophoblastic cells. This characteristic makes miR-21 a promising candidate for targeted approaches in the diagnosis and treatment of gestational trophoblastic neoplasms [79]. 

*Twist-1*, a negative regulator of E-cadherin, shows significantly elevated levels in CHM and correlates with disease invasiveness [75,84,85]. E-cadherin, crucial for cell-to-cell adhesion, exhibits reduced expression associated with GTD and is associated with increased invasiveness [14,74,75,84], although one study with a small sample failed to show a significant association [74]. 

Thiol group-containing compounds, such as glutathione, are ROS scavengers. The thiol group (–SH) can donate a hydrogen atom in redox reactions to reduce oxidative stress, which results in the formation of disulfide bonds (–S–S–) [107]. Thus, a decreased thiol/disulfide ratio indicates a higher oxidative stress state. Peckan et al. demonstrated a decreased thiol/disulfide ratio in molar pregnancy compared to non-molar placental tissue, indicating higher oxidative stress in molar pregnancy. Similarly, Incebiyik et al. showed that markers for both oxidative stress and apoptosis (M30) were higher in CHM than normal pregnancy and parallel to each other [108]. Oxidative stress is generally higher during pregnancy and can be even more pronounced in pregnancy complications such as pre-eclampsia [109]. An imbalance between antioxidant capacity and reactive oxygen species (ROS) can lead to oxidative damage in the maternal and placental compartments [109].

Since the 1980s, numerous studies have demonstrated that GTD, particularly choriocarcinoma, and pre-eclampsia share common pathogenic mechanisms, including disruptions in angiogenesis, increased oxidative stress, and complement dysregulation [109,110,111,112]. Both pre-eclampsia and choriocarcinoma exhibit abnormalities in angiogenesis regulation. In pre-eclampsia, there are elevated levels of the antiangiogenic protein sFlt-1 (Soluble Fms-like Tyrosine Kinase 1) and reduced levels of angiogenic factors such as vascular endothelial growth factor (VEGF) and placental growth factor (PlGF) [110]. Conversely, choriocarcinoma is associated with increased levels of the pro-angiogenic factor PlGF [110]. Given these parallels, mechanisms proven to be involved in pre-eclampsia warrant investigation in GTD. For instance, CD93, an angiogenic factor known to play a significant role in pre-eclampsia, has yet to be studied in the context of molar pregnancies [113]. Future research exploring the role of CD93 in GTD could reveal new potential therapeutic targets.

## 4. Conclusions

In conclusion, the molecular landscape of molar pregnancies unveils a complex interplay of genetic and epigenetic factors that influence disease progression and clinical outcomes. The overexpression of p53 in cytotrophoblasts and dysregulation of its modulators, ASPP1/2 and iASPP, highlights the proliferative potential and invasive nature of molar tissues. Moreover, discrepancies in proteins involved in apoptotic pathways such as BCL-2 and caspases highlight the dysregulation of the balance between cell survival and death in GTDs. The differential expression of growth factor receptors such as EGFR, ERBB2, and CD117 in trophoblasts reflects their roles in promoting cell proliferation and invasion, contributing to the aggressive behavior observed in some molar pregnancies. 

Despite these advancements, challenges remain in standardizing diagnostic criteria and therapeutic approaches for molar pregnancies. Future research should focus on elucidating the specific molecular pathways driving molar pregnancy pathogenesis and developing targeted interventions to improve clinical outcomes. It may be specifically helpful in preventing the recurrence of GTD and GTNs. By integrating molecular insights with clinical observations, we can advance our understanding of GTDs and enhance patient care through personalized medicine strategies.

## Figures and Tables

**Table 1:** Genetic variants and mutations associated with Recurrent Hydatidiform Mole (RHM).

Chromosome Number	Mutation [Reference]	Gene Involved
1	c.[1796T>A]; [1796T>A] [62]	PADI6
6	6p21.33 Mutation p.M1V, c.1A>G [52]	KHDC3L
	c.322_325delGACT [6,15,16,17] c.602 C>G [48] c.299_302delTCAA, p.Ile100Argfs*2 c.322_325delGACT, p.Asp108Ilefs*30 [47] c.17_20delGGTT, p.Arg6Leufs*7 c.349+1G4A [53] c.232C>T, p.Arg78Ter [56]	
6	c.334 1G>A [67]	REC114
11	pUPD 11p15.4 [63]	TH01
11	c.783dup (p.Glu262*) missense variant c.1501T>C (p.Ser501Pro) [67]	TOP6BL/C11orf80
13	G48C(p.Q16H) [64]	ERC1c
16	c.G1114A(p.G372S) [64]	KCNG4
17	Exon6-213(nonsense) p.Arg213X Exon6-220(missense) p.Tyr220Cys Exon7-245(missense) p.Gly245Ser Exon7-248(missense) p.Arg248Gln Exon7-249(missense) p.Arg249Ser Exon8-295(missense) p.Pro295Leu [68]	TP53
19	c.1441G>A [48] Exon 4 (missense) c.1358T>G, p.Ile453Ser Exon 7 (Frameshift) c.2655dupC, p.Ile886HisfsTer11 [54] c.555_557delCAC, p.Thr185del [55] c.2810+2T>G [58] Exon 2, c.197G>A [65] c.584G>A; p.W195X [66] Exon 6/intron 9 (c.[2248C4G]; [2810+2T4G]) Exon 4 (c.[1374_1375delAG]; [1374_1375delAG]) Exon 4/6 (c.[1908dup]; [2161C4T]) Exon4/intron 10 (c.[939_952dup14]; [2982-2A4G]) Exon 9 (c.[2759G4A]; [2759G4A]) Exon 9 (c.[2777T4G]; [2777T4G]) Intron 5 (c.[2130-6_2132del]; [ = ]) Intron 5 & 6 (c.[2130-266_2300+782del]; [2130-266_2300+782del]) Before exon 1 & intron 5 (c.[ −39-231_2130-510del]; [-39-231_2130-510del]) Intron 1 & intron 5/exon 8 (c.[-40+251_2130-681del];[2571dup]) Intron 1 & intron 5 (c.[-3998_2130-668del]; [-3998_2130-668del]) Before exon 1/exon 6 (c.[-13413_2982-344del];[2248C4G]) [61] c.[1812_1837dup]; [1812_1837dup] c.[2162G>A]; [2162G>A] c.[2204A>C]; [2204A>C] c.[−40 + 3G > C]; [−40 + 3G > C] c.[−6831_-39–1586]; [2248C > G] [62] c.1093G > A, p.(Asp365Asn) c.[1093G > A]; [1093G > A] [62]	NLRP7
22	c.3452G>A c.1196þ1G>A, affecting the splice donor of exon 10, and a 1-bp deletion, c.2206del (p.Val736Serfs*31), in exon 19 [67]	MEI1

**Table 2:** Molecular dysregulation in GTD.

Gene	Reference	Detection Method	GTD Diagnosis Method	Result
BCL-2	[68,71,73]	TMA; IHC; IHC	Morphological appearance and p57 IHC; Morphological appearance and ploidy analysis by flow cytometry; Morphological appearance and STR genotyping	Decreased expression in CHM compared to PHM and control
BCL-2	[72]	IHC	Morphological appearance and p57 IHC nuclear DNA microsatellite polymorphism for discordant cases	Increased expression in CHM and PHM compared to HA
CD117	[73]	IHC	Morphological appearance and STR genotyping	Decreased expression in HA compared to CHM and PHM
CD44v6	[74]	IHC	Not specified	No significant difference in CD44v6 expression
c-erB-2	[73]	IHC	Morphological appearance and STR genotyping	Increased expression in CHM compared to PHM and HA
E-cadherin	[14,75]	IHC; IHC	Morphological appearance and molecular genotyping; Morphological appearance, ploidy analysis by flow cytometry, and p57 IHC	Decreased expression in HM compared to HA
IGF-1	[76]	IHC	Morphological appearance and p57 IHC	Downregulation in CHM decidua and chorionic villi
Inhibin-alpha	[14]	IHC	Morphological appearance and molecular genotyping	Increased expression in HM compared with HA
Ki-67	[72,75,77]	IHC for all	Morphological appearance and p57 IHC; Morphological appearance, ploidy analysis by flow cytometry, and p57 IHC; Morphological appearance	Increased expression in CHM compared to PHM and HA. Increased expression in PHM compared to HA.
LIF	[76]	IHC	Morphological appearance and p57 immunostaining	Downregulated in CHM decidua but upregulated in CHM trophoblasts.
MAP3K1	[78]	IHC	Not specified	Significantly higher expression in CHM compared to control
miRNA-21	[79]	qRT-PCR	Morphological appearance	miRNA-21 is upregulated in HM
miR-196b	[78]	qRT-PCR	Not specified	Significantly lower expression in CHM compared to control.
P53	[14,72,80,81]	IHC; IHC; IHC; IHC	Morphological appearance and molecular genotyping; Morphological appearance and p57 immunostaining; Morphological appearance; Morphological appearance and ploidy analysis by flow cytometry	Increased expression in CHM compared to PHM and HA. Increased expression in PHM compared to HA.
P57	[73,82]	IHC; IHC	Morphological appearance and STR genotyping; Not specified	Absent expression in all CHM (both androgenetic diploidy and biparental diploidy) No significant difference in expression between PHM and HA
P63	[72,83]	IHC; IHC	Morphological appearance, p57 immunostaining, and nuclear DNA micro- satellite polymorphism for discordant cases; Morphological appearance	Increased expression in CHM and PHM compared to HA
Twist-1	[75,84,85]	IHC; IHC; IHC	Morphological appearance, ploidy analysis by flow cytometry, and p57 immunostaining; Morphological appearance and p57 immunostaining; Morphological appearance and p57 immunostaining and ploidy analysis	Expression is significantly higher in CHM compared to PHM and HA

BCL-2 = B cell lymphoma-2, TMA = Tissue Microarray, PMH = Partial hydatidiform mole, CHM = Complete hydatidiform mole, IHC = Immunohistochemistry, HA = Hydropic abortion, HM = Hydatidiform mole, STR = Short tandem repeat, miRNA = Micro-Ribonucleic acid, qRT-PCR = quantitative Reverse Transcription Polymerase Chain Reaction, quantitative real-time polymerase chain reaction

## Data Availability

No new data were created or analyzed in this study. Data sharing is not applicable to this article.

## Supplementary Materials

The following supporting information can be downloaded at: https://www.mdpi.com/article/10.3390/ijms25168739/s1.
