# Supplementary material for: Molecular Basis of Hydatidiform Moles—A Systematic Review
Source: Int J Mol Sci. 2024 Aug 10;25(16):8739. doi: 10.3390/ijms25168739 (PMC11354253; doi:10.3390/ijms25168739)
Supplement: Supplementary file 1 [file ijms-25-08739-s001.zip › ijms-3118643-supplementary.pdf]

Supplementary table S1: Details of the included studies

	Author	Year	Type of study	Sample size	Main finding	Reference number
1.	Rath et al.	2023	Case report	A female patient with four consecutive complete molar pregnancies	A homozygous variant of KHDC3L gene was detected (chr6:g.73363157C>T) which results in a stop codon and premature truncation of the protein at codon 78 (p.Arg78Ter)	[56]
2.	Al-Jabri et al.	2023	Cross-sectional	95 PHM<br>142 CHM	Significantly lower BCL-2 expression in CHM compared to PHM	[70]
3.	Jahanbin et al.	2023	Cross-sectional	40 PHM<br>47 CHM	Significantly higher expression of Twist-1 in villous stromal cells of CHM compared to PHM	[92]
4.	Xing et al.	2022	Retrospective	21 cases of twin/multiple gestations with a molar component, including 20 CHMs (17 twins, 2 triplets, 1 quintuplet) and 1 PHM (twin).	All CHM components were p57-negative and those genotyped were purely androgenetic.	[27]
5.	Hadi et al.	2022	Cross-sectional	32 PHM<br>24 CHM<br>4 IM<br>2 CC	Expression of p53 was significantly associated with IM and CC ($p < 0.001$)	[89]
6.	Zainal et al.	2021	Cross-sectional	41 PHM<br>39 CHM<br>2 Unclassified HM	Discordance between routine H&E and p57kip2 IHC of 33.0%	[28]
7.	Ndukwe et al.	2021	Cross-sectional	33 PHM<br>21 CHM	Discordance between routine H&E diagnosis and p57kip2 IHC staining in 8 cases	[21]
8.	Rezaei et al.	2021	Case series	9 patients with RHM	Seven functional variants in a recessive state associated with RHM:<br>Five variants in NLRP7<br>One variant each in NLRP5 and PADI6	[62]
9.	Xing et al.	2021	Cross-sectional	2,217 cases, including 2,160 uterine and 57 ectopic specimens.	CHMs were predominantly p57-negative (99.8%) and genotypically androgenetic (96.7%).	[25]

					PHMs showed predominant p57-positive expression (99%) and genotypically were mostly diandric triploid (97%).	
10	Lin et al.	2021	Prospective Cohort	39 complete moles	Identification of a distinct microRNA profile (miR-181b-5p and miR-181d-5p) associated with complete moles progressing to gestational trophoblastic neoplasia.	[73]
11	Pekcan et al.	2020	Case-control study	41 HM<br>41 Control	Significant decrease in thiol level in HM	[107]
12	Alici-Garipcan et al.	2020	Experimental in vitro	Human skin samples collected from a HM patient with impaired NLRP7 expression and a healthy volunteer	Impaired NLRP7 expression results in downregulation of pluripotency factors, activation of trophoblast lineage markers, and maturation of the extraembryonic cell types.<br><br>BMP pathway inhibition corrected the excessive trophoblast differentiation of patient-derived Pluripotent stem cells	[86]
13	Zhang et al.	2020	Experimental in vitro	12 patients with NLRP7 NSVs	NLRP7 NSVs affect the processing and secretion of IL-1β in patients with RHM.	[59]
14	Fallahi et al.	2020	Case report	1 patient with history of 5 HM	Identification of a homozygous mutation (p.M1V, c.1A > G) in the KHDC3L gene in the patient with RHM	[52]
15	Fallahi et al.	2020	Case series	14 Iranian patients with history of RHM	Identification of a specific mutation (c.1A>G) in the KHDC3L gene in patients with RHM	[51]
16	Zheng et al.	2020	Prospective Cohort	165 CHM:<br>138 homozygous<br>27 heterozygous	Heterozygous/dispermic complete moles are clinically more aggressive and have a significantly higher risk for developing post-molar GTD compared to homozygous/monospermic CHM ($p = 0.0009$)	[16]
17	Nagib et al.	2019	Cross-sectional study	24 CHM	The level of expression of Ki-67 and Caspases has no association with disease persistance	[99]

18	Deka et al.	2019	Cross-sectional	30 HA<br>30 PHM<br>30 CHM	Significantly higher Ki-67 expression in cytotrophoblasts in CHM than PHM than HA<br><br>Significantly higher Ki-67 expression in stromal cells in molar pregnancy than HA	[82]
19	Shalabi et al.	2019	Case report	40-year-old Egyptian woman with RHM and CC	Two mutations identified in NLRP7 c.1358T>G, c.2655dupC	[54]
20	Kar et al.	2019	Prospective case control	48 GTD<br>8 HA<br>40 normal placentas	Cyclin E and Ki-67 showed stronger staining intensity in CHM, CC, and PSTT.	[97]
21	Khooei et al.	2019	Case-control	10 HA<br>8 PHM<br>11 CHM	Significantly higher p53 expression in HM compared to HA	[88]
22	Missaoui et al.	2019	Case control	39 HA<br>41 PHM<br>140 CHM	Increased expression of BCL-2 in CHM and PHM compared to HA ($p < 0.0001$ and $p = 0.001$ respectively)<br><br>Increased expression of ki-67 in CHM compared to PHM and HA ($p = 0.005$)<br><br>Increased expression of p53 in CHM compared to PHM and HA ($p < 0.0001$)<br><br>Increased expression of p63 in CHM and PHM compared to HA ($p = 0.0001$ and $p = 0.001$ respectively)	[74]
23	Khooei et al.	2019	Case-control	10 HA<br>8 PHM<br>11 CHM	Decreased expression of BCL-2 in CHM compared to PHM and HA	[72]
24	Takahashi et al.	2019	Experimental in vitro		Decreased or absent induction of p57KIP2 was associated with reduced sensitivity of TSmole cells to contact inhibition.	[29]
25	King et al.	2019	Case-control	26 samples of CHM from 23 patients<br>29 control	Abnormalities in epigenetic pathways were identified in CHMs, specifically in DNA methylation and	[32]

					imprinting patterns including downregulation of DNMT3A	
26	Buza et al.	2019	Case series	3 cases of HM	Paternal uniparental isodisomy of the tyrosine hydroxylase locus at chromosome 11p15.4 can lead to abnormal gestations that mimic hydatidiform mole both clinically and histologically.	[63]
27	Fallahi et al	2019	Case study	A woman with 5 RHM and her sister with miscarriage	A novel mutation in the NLRP7 gene (c.555_557delCAC, p.Thr185del) was identified in homozygous state in the patient with recurrent molar pregnancies and a heterozygous state in her sister.	[55]
28	Ji et al	2019	Case-control	5 HM<br>5 control	NLRP7 c.1441 G>A mutation was associated with biparental complete moles only.	[48]
29	Guo et al.	2019	Case-control	20 CHM<br>15 control	Significantly lower expression of miRNA-196b in CHM compared to control<br><br>Significantly higher expression of MP3K1 in CHM compared to control.	[83]
30	Chan et al.	2019	Observational/ Experimental in vitro	10 First trimester placentae<br>11 Term placentae<br>63 HM<br>7 CC	iASPP is overexpressed in HM and CC compared to normal placenta<br><br>Overexpression of iASPP was associated with increased autophagy related protein expression while its silencing was associated with cellular senescence	[80]
31	Cicek et al	2018	Case control	8 PHM<br>8 CHM<br>8 control	IGF-1 expression is downregulated in CHM decidua and chorionic villi.<br><br>LIF expression is downregulated in CHM decidua but upregulated in CHM trophoblasts.	[81]

32	Moussa et al	2018	Case-control	16 HA<br>17 PHM<br>16 CHM	Significantly decreased E-cadherin expression in HM compared to HA<br><br>Significantly increased Ki-67 expression in PHM compared to HA<br><br>Twist-1 expression is significantly higher in CHM compared to PHM and HA	[79]
33	Nguyen et al.	2018	Case-control	MEI1 and REC114 were screened in 99 affected women<br><br>TOP6BL/C11orf80 was screened in 246 affected women	Identification of genetic mutations in MEI1, TOP6BL/C11orf80, and REC114<br>associated with recurrent androgenetic CHM.	[68]
34	Nguyen et al.	2018	Cross-sectional	113 patients with RHM	Mutations in NLRP7 and KHDC3L were associated with diploid biparental HM, while recurrent molar pregnancies without mutations were associated mostly with diploid androgenic monospermic and triploid biparental dispermic	[50]
35	Chan et al.	2018	Cross-sectional	49 HM	P53 mutations were identified: two missense mutations (p.R249S and p.R248Q) that disrupt p53 DNA binding sites, and a nonsense mutation (p.R213X) that prematurely truncates the protein, resulting in loss of function	[65]
36	Zhao et al.	2018	Experimental in vitro	Control 6<br>Regressed CHM 35<br>Post-CHM GTN 21	miR-371a-5p and miR-518a-3p were upregulated in progressed CHMs (GTN)<br><br>Functional analyses showed that miR-371a-5p and miR-518a-3p promoted proliferation,	[15]

					migration, and invasion of choriocarcinoma cells	
37	Kubelka et al.	2017	Case series	8 CHM	Absent expression of p57 in all CHM (both androgenetic diploidy and biparental diploidy)	[90]
38	Khashaba et al	2017	Cross-sectional	11 PHM<br>45 CHM	p57Kip2 IHC reclassified seven cases as CHM and one case as PHM.	[22]
39	Samadder et al.	2017	Cross-sectional	23 CHM<br>4 PHM<br>1 unclassified HM<br>25 controls	Negative immunostaining of p57Kip2 in 96% of CHM cases	[41]
40	Lelic et al.	2017	Cross-sectional	12 CHM<br>185 PHM<br>1 unclassified HM	p57 immunostaining had 100 % concordance with pathohistological diagnoses in CHM group but 92% concordance in PHM group.	[23]
41	Kheradmand et al.	2017	Case-control	20 PHM<br>20 HA	Rate and intensity of stating was higher in PHM compared to HA ($p = 0.027$ and $p < 0.001$ respectively)	[87]
42	Wang et al	2017	Experimental in vitro	16 HM<br>20 normal placenta	miR-21 expression was significantly higher in HM tissues compared to control ($p< 0.05$).<br><br>miR-21 inhibition significantly inhibited cell proliferation in choriocarcinoma cell lines ($p <0.05$), and overexpression promoted migration, and invasion in choriocarcinoma cell lines ($p< 0.01$ and < 0.05 respectively)	[85]
43	Sills et al.	2017	Case report	One patient with RHM and a homozygous pathogenic variant in NLRP7 (c.2810+2T > G) who underwent IVF	all embryos (total 10) from the patient arrested in development by 144 hours in culture.<br><br>Karyomapping of the non-viable embryos revealed that all were diploid biparental. 8 embryos had variable aneuploidies.	[58]
44	Yu et al.	2017	Case-control	WES was done for 51 CHM patients	Two SNPs were associated with an increased risk of CHM (p	[64]

				and 47 healthy women. Candidate variants were analyzed in 199 CHM patients and 400 healthy controls	< 0.05): c.G48C (p.Q16H) in the ERC1 gene and c.G1114A (p.G372S) in the KCNG4 gene	
45	Incebiyik et al.	2016	Case- control study	34 CHM<br>34 Control	Higher M30 and M65 (apoptosis markers) and Oxidative stress index in CHM	[108]
46	Triratanachat et al.	2016	Cross-sectional	97 CHM<br>30 PHM	P57KIP2 IHC results were discordant in 12 cases (9.4%) with the histopathological diagnosis.	[40]
47	Erol et al.	2016	Case-control	17 HA<br>23 PHM<br>20 CHM	Increased BCL-2 expression in HA compared to CHM and PHM ($p < 0.001$).<br>Decreased CD117 staining percentage in HA compared to CHM and PHM ($p < 0.001$).<br>Increased c-erB-2 expression in CHM compared to PHM and HA ($p = 0.003$)<br>Absent expression of p57 in CHM<br>No significant difference between PHM and HA ($p < 0.001$)	[75]
48	Erol et al.	2016	Case-control	23 HA<br>24 PHM<br>23 CHM	Decreasing E-cadherin expression from HA to PHM to CHM ($p < 0.001$)<br><br>Increased inhibin-alpha expression in molar pregnancy compared with HA ($p < 0.001$)<br><br>Increased expression of p53 in CHM compared to PHM and HA ($p < 0.001$)	[14]
49	Hasanzadeh et al.	2016	Cross-sectional	10 PHM<br>18 CHM<br>30 GTN	Increased c-erB-2 expression in cytotrophoblasts in GTN compared to simple HM ($p = 0.000$).	[78]

					Increased expression of p53 in GTN compared to simple HM (p = 0.000).	
50	Bolze et al.	2016	Case-control	8 Control<br>6 PHM<br>12 CHM<br>1 IM<br>1 CC<br>1 PSTT	the staining intensity of the Syncyntin-1 surface subunit C-terminus was significantly higher in HM, especially those with malignant transformation on follow up (p < 0.001)	[36]
51	Sun et al.	2016	Case control	Control 48<br>Regressed HM 49<br>Progressed HM 39	Maspin was inversely correlated with FIGO prognostic score (p = 0.041) whereas expression of m-p53 was positively correlated with FIGO stage (p= 0.019).	[84]
52	Braga et al.	2016	Retrospective cohort	Regressed CHM 590<br>Post-CHM GTN 190	The NPV for GTN of apoptotic index (using Capase-3 IHC staining) ≥ 4.0% was 97%	[76]
53	Wang et al.	2016	Case control	Control 36<br>PHM 25<br>CHM 48<br>IM 12	Decreasing IMP3 expression from normal placental tissues, to PHM, to CHM, to IM (p < 0.05)	[77]
54	Hemida et al.	2016	Case report	Egyptian woman with FRHM	Sequencing of the NLPR7 gene in the patient revealed a homozygous base change in exon 2, c.197G>A, leading to a truncated protein p.W66∗.	[66]
55	Ito et al.	2016	Case series	four Japanese RHM cases	Whole-exome sequencing identified a homozygous nonsense mutation in the NLRP7 gene (c.584G>A; p.W195X) in one patient.<br><br>Genotyping of molar tissues confirmed biparental origin in all four cases.<br><br>There was a specific loss of maternal DNA methylation in DMRs of PEG3, SNRPN, and PEG10.	[67]
56	Rezaei et al.	2016	Case series	One Iranian patient and one Indian patient with RHM	Identified a homozygous 4-bp deletion mutation in KHDC3L (c.17-20delGGTT; p.Arg6Leufs∗7) in the Iranian patient and a	[53]

					homozygous splice mutation in KHDC3L (c.349+1G>A) in the Indian patient.<br><br>No mutation in NLRP7 gene was found.	
57	Reddy et al.	2016	Case series	16 patients with RHM	11 Novel NLRP7 variants were identified	[60]
58	Rahat et al.	2016	Cross sectional	30 first trimester normal pregnancy<br>30 second trimester normal pregnancy<br>30 third trimester normal pregnancy<br>30 pregnancy complicated with pre-eclampsia<br>15 molar pregnancy	Development of choriocarcinoma was associated with DNA methylation and associated with lower expression of STAT5.	[39]
59	Luchini et al.	2015	Case control	23 Abortions<br>10 PHM<br>12 CHM<br>7 Term placentae	Expression of twist-1 is significantly higher in CHM compared to PHM ($p < 0.05$) and HA ($p < 0.001$)	[93]
60	Fock et al	2015	Case-control	12 CHM<br>50 Healthy placentae<br>5 healthy decidua	Trophoblasts with invasive characteristics have significantly increased expression of ERBB2 and ERBB3	[104]
61	Wargasetia et al.	2015	Case control	6 Control<br>11 PHM<br>11 CHM<br>11 IM<br>9 CC	Decreasing BCL-2 expression from PHM, to CHM, to invasive mole, to choriocarcinoma compared to normal placenta ($p< 0.002$)<br><br>Increased Beclin-1 expression in choriocarcinoma ($p < 0.05$)	[71]
62	Pasdar et al	2015	descriptive observational study	20 HM<br>20 non-molar pregnancies	CHM: 9 out of 10 cases analyzed were diploid, and 1 case was tetraploid. PHM: 8 out of 10 cases analyzed were triploid, and 2 cases were diploid.	[20]

					Spontaneous Abortions: All 20 were diploid.	
63	Masood et al.	2015	Case control	HA 30<br>PHM 30<br>CHM 30	Increased intensity of p63 staining in HM compared to HA ($p < 0.001$)	[91]
64	Sanchez et al.	2015	Cross sectional	4 androgenetic moles<br>5 RHM with NLRP7 mutation	Lack of methylation at maternal DMRs, might be associated with the development of RHM in patients with NLRP7 mutations.	[35]
65	Lertkhachonsuk et al.	2015	Observational/ Cohort	1- Compared LINE-1 Methylation in:<br>12 control<br>38 HM<br>19 GTN<br><br>2- For the longitudinal study: 145 hydatidifo-rm mole patients	Significant increase in unmethylated LINE-1 loci in the malignant trophoblast group compared to hydatidiform moles.<br><br>Lower level of partially methylated LINE-1 loci and partially unmethylated LINE-1 loci were associated with a higher risk of developing postmolar GTN.<br><br>When methylation level is combined with pretreatment β-hCG levels, the predictive accuracy for GTN, has a PPV of 77.4% and a NPV of 83.8%	[38]
66	Chen et al.	2014	Retrospective	15 CHM<br>11 PHM<br>15 HA	p57 Staining was negative in<br>all CMs and positive in all PMs and HAs	[26]
67	NM et al.	2014	Experimental	36 HM from patients with two defective alleles in the NLRP7 gene.	All POCs were diploid biparental. Missense mutations were associated with positive p57KIP2 and features of partial HMs. Protein-truncating mutations were linked to negative p57KIP2 and characteristics of complete HMs. Severe mutations caused excessive proliferation; mild mutations allowed some differentiation.	[61]
68	Sasaki	2014	Retrospective observational	14 equivocal cases were	p57kip2 IHC successfully differentiated CHM	[24]

				stained with p57kip2 and staining compared to stained sections of DNA established androgenetic CHM, triploid PHM and biparental abortions	(negative staining) from PHM or HA (positive staining) in all 14 cases.	
69	Zheng et al.	2014	Cross sectional	146 cases of suspected or diagnosed molar pregnancies underwent STR DNA genotyping	95 cases classified as CHM (92 monospermic and 3 dispermic)<br><br>34 cases classified as PHM (32 dispermic and 2 monospermic)<br><br>17 cases classified as balanced biallelic gestations.	[30]
70	Banet et al.	2014	Cross sectional	201 CHM<br>158 PHM<br>272 non-molar<br>14 androgenetic/biparental mosaics.	199 cases of complete moles were p57-negative. 1 was non-reactive and 1 was p57 positive androgenetic with retained maternal copy of chromosome 11<br><br>156 of PHM were p57-positive. And 2 PHM were p57-negative due to loss of maternal copy of chromosome 11.<br><br>Non-molar specimens included 259 p57-positive biparental diploid cases, 9 p57-positive digynic triploid cases, and 2 p57-negative biparental diploid cases without morphological features of biparental hydatidiform mole and an uncertain etiology for loss of p57 expression.	[31]
71	Mahadevan et al.	2014	Experimental in vitro	Human Embryonic Stem Cells	NLRP7 interacts with YY1, an important chromatin-binding factor and can alter DNA	[34]

					methylation affecting trophoblast differentiation.	

PHM= Partial hydatidiform mole, CHM= Complete hydatidiform mole, BCL-2= B cell lymphoma-2, IM= Invasive mole, CC= Choriocarcinoma, HM= Hydatidiform mole, H&E= Hematoxylin and eosin, IHC= Immunohistochemistry, GTN= Gestational trophoblastic neoplasia, SD= Standard deviation, RHM= Recurrent hydatidiform mole, NLRP7= NLR family pyrin domain containing 7, NLRP5= NLR family pyrin domain containing 5, PADI6= Peptidylarginine deiminase 6, BMP= Bone Morphogenetic Protein, NSVs= Non synonymous variants, IL-1β= interleukin-1β, KHDC3L= KH Domain Containing 3 Like, HA= Hydropic abortion, GTD= Gestational trophoblastic disease, PSTT= Placental site trophoblastic tumor, TSmole cells= Trophoblast stem cells from hydatidiform moles, DNMT3A= DNA methyltransferase 3 alpha enzyme, iASPP= inhibitor of apoptosis-stimulating protein of p53, IGF-1= Insulin like growth factor-1, LIF= leukemia inhibitory factor, IVF= In vitro fertilization, WES= Whole-exome sequencing, SNPs= single nucleotide polymorphisms, KCNG4= potassium voltage-gated channel modifier subfamily G member 4, FIGO= International Federation of Gynecology and Obstetrics, NPV= Negative predictive value, Apoptotic index = Positive caspase -3 staining cells / negative caspase-3 staining cells x 100, IMP3= insulin-like growth factor II mRNA-binding protein 3, FRHM= Familial recurrent hydatidiform mole, DMRs= differentially methylated regions, PEG3 = Paternally Expressed Gene 3, SNRPN = Small Nuclear Ribonucleoprotein Polypeptide N, PEG10 = Paternally Expressed Gene 10, STAT5= Signal Transducer and Activator of Transcription 5, ERBB2 = Receptor Tyrosine-Protein Kinase erbB-2, ERBB3 = Receptor Tyrosine-Protein Kinase erbB-3, LINE-1= Long Interspersed Nuclear Element-1, β-hCG= beta human chorionic gonadotropin, PPV= Positive predictive value, STR= Short tandem repeat, YY1= Yin Yang 1
